# Target Enzymes of *Origanum majorana* and *Rosmarinus officinalis* Essential Oils in Black Cutworm (*Agrotis ipsilon*): In Vitro and In Silico Studies

**DOI:** 10.3390/insects15070483

**Published:** 2024-06-28

**Authors:** Fatma S. Ahmed, Walid S. Helmy, Nawal Abdulaziz Alfuhaid, Moataz A. M. Moustafa

**Affiliations:** 1 Department of Economic Entomology and Pesticides, Faculty of Agriculture, Cairo University, Giza 12613, Egypt; fatma.sherif@cu.edu.eg (F.S.A.); wshelmy@cu.edu.eg (W.S.H.); 2 Department of Biology, College of Science and Humanities, Prince Sattam Bin Abdulaziz University, Al-Kharj 11942, Saudi Arabia; n.alfuhaid@psau.edu.sa

**Keywords:** essential oils, *A. ipsilon*, α-esterase, GST, ATPases, in silico, molecular docking

## Abstract

**Simple Summary:**

The black cutworm, *Agrotis ipsilon* (Hufnagel), poses a significant threat to various crops. Marjoram (*Origanum majorana*) and rosemary (*Rosmarinus officinalis*) essential oils (EOs) were investigated for their toxicity using in vitro and in silico methods. GC-MS analysis identified the main constituents, with *O. majorana* being more toxic than *R. officinalis* to *A. ipsilon* larvae. *R. officinalis* EO inhibited Na^+^/K^+^ pump activity consistently, while *O. majorana* showed varied effects. Both EOs influenced detoxification enzymes differently over time. Molecular docking indicated a strong binding affinity of the main EO constituents to target enzymes. These findings suggest the potential of EOs as insecticides in integrated pest management programs, particularly in organic agriculture.

**Abstract:**

In this study, in vitro and in silico approaches were employed to assess the toxicity of marjoram (*Origanum majorana*) and rosemary (*Rosmarinus officinalis*) essential oils (EOs) to *A. ipsilon* larvae. The study determined the activities of ATPases in the larvae after treatment with the LC_20_ and LC_70_ of each EO. α-esterase and glutathione-S-transferase (GST) activities were also determined after treatment with LC_10_ and LC_30_ of each EO. Furthermore, molecular docking was employed to determine the binding affinity of terpinene-4-ol and α-pinene, the major constituents of *O. majorana*, and *R. officinalis* EOs, respectively, compared to the co-crystallized ligand of α-esterase, diethyl hydrogen phosphate (DPF). Toxicity assays revealed that *O. majorana* EO was more toxic than *R. officinalis* EO to the *A. ipsilon* larvae at 96 h post-treatment. However, the LC_20_ and LC_70_ of the latter significantly inhibited the activity of the Na^+^-K^+^ pump at almost all intervals. The same concentrations significantly inhibited the Mg^2+^/Ca^2+^-ATPase and Ca^2+^ pump at 96 h post-treatment. In contrast, *O. majorana* EO showed a variable effect on the Na+-K+ pump across different time intervals. On the other hand, LC_10_ and LC_30_ of both EOs showed varied effects on α-esterase and GST over time. Molecular docking revealed energy scores of −4.51 and −4.29 kcal/mol for terpinene-4-ol and α-pinene, respectively, compared to a score of −4.67 for PDF. Our study demonstrated the toxicity of the tested EOs to *A. ipsilon*, suggesting their potential efficacy as insecticides.

## 1. Introduction

*Agrotis ipsilon*, the black cutworm, is a globally recognized insect pest of significant agricultural concern. Its impact is notable, particularly in Egypt, where it has garnered substantial research interest due to the extensive damage it inflicts on a variety of such crucial vegetable and field crops as corn, cotton, soybeans, beans, potatoes, clover, and tomatoes [1]. Historically, the management of this pest has been heavily reliant on the use of insecticides [2]. However, the exclusive dependence on insecticides as a rapid solution for pest eradication poses such challenges as resistance, secondary pest outbreaks, and health and environmental issues [3,4]. These challenges have sparked interest in exploring naturally occurring compounds as alternatives for pest management [5,6,7,8]. Recent studies have provided evidence that plant EOs could serve as an effective tool in managing this destructive pest [9,10,11,12,13,14]. This shift towards more natural pest control represents a significant advancement in our approach to sustainable agriculture.

Plant EOs are produced as secondary metabolites within the secretory structures of plant organs. Based on their synthesis, these metabolites are classified into two chemical groups (i) terpenoids, and (ii) phenylpropanoids [15]. Both groups have exhibited enormous potential as acute or chronic insecticides [6], insect growth regulators [16], or antifeedants [17,18] against a variety of insect species. Such effects may be correlated with the magnitude of biochemical changes in the test species. For example, [19] revealed that *Lavandula multifida* EO affected the detoxification enzymes α-esterase and glutathione-S-transferase (GST) of *A. ipsilon* and *Spodoptera littoralis* at 96 h post-treatment. Study [20] also reported that *Cymbopogon citratus* EOs inhibited the carboxylesterases (CarEs) and GST enzymes of *A. ipsilon* larvae. Study [21] reported that *Arisaema fargesii* EOs significantly affected the *α*-esterase, *β*-esterase, p-nitrophenyl acetate (p-NPA) esterase, and GST of *Aedes aegypti* larvae. Furthermore, numerous studies have validated the inhibitory potential of EOs on insect cytochrome P450s, CarEs, and GSTs establishing these enzymes as prospective target sites in insects [4,22]. Nevertheless, the precise mechanisms underlying the enzyme inhibition by EOs remain unclear [23].

Adenosine triphosphate-hydrolyzing enzymes (ATPases) are transmembrane proteins that play a key role in a varied array of cellular functions across all kingdoms of life [24]. These dynamic proteins transport solutes across membranes and act as molecular motors that use the energy of ATP hydrolysis to conduct such mechanical works as ion pumping, cellular metabolism, muscle movement, protein trafficking, unfolding, replication, and transcription [25]. These transmembrane proteins were reported to be target sites for insecticides such as DDT, chlorpyrifos, beta cypermethrin, abamectin, thiamethoxam, and diafenthiuron [26,27,28]. In addition, some reports have revealed that some plant compounds have insecticidal effects due to their inhibition of ATPases [29,30]. However, to our knowledge, little or no data are available on the effect of *Origanum majorana* and *Rosmarinus officinalis* EOs on the activities of ATPases such as Na^+^/K^+^-ATPase (Na^+^/K^+^-pump), Mg^2+^/Ca^2+^-ATPase, and Ca^2+^-ATPase (Ca^2+^ pump) in *A. ipsilon*. 

Marjoram (*O. majorana*) and rosemary *(R. officinalis*) are perennial aromatic herbs (family Lamiaceae) native to the Mediterranean regions [31,32,33]. Numerous studies have confirmed the toxicity of *O. majorana* EOs [31,34,35,36,37,38] and *R. officinalis* EOs [33,39,40,41,42,43,44,45,46] to various pests. However, the effect of these EOs on α-esterase and GST activity in *A. ipsilon* has not been well investigated.

In the realm of drug discovery, in silico methods play a pivotal role by enabling the virtual screening of millions of compounds within a short timeframe [47]. This approach significantly reduces the initial costs associated with compound identification and enhances the likelihood of identifying promising drug candidates. Currently, a diverse array of molecular modeling techniques exists to facilitate drug discovery. These methods are primarily categorized based on their structural and ligand-based approaches [48] and one of the widely used techniques is *molecular docking*, which predicts the molecular orientation of a ligand within the receptor. Subsequently, the replacement of the ligand in the receptor is estimated using a scoring function [49].

The current study investigated the chemical composition of *O. majorana* and *R. officinalis* EOs and their insecticidal activity on *A. ipsilon* larvae. The primary objective is to delve into the effects of *O. majorana* and *R. officinalis* EOs on the ATPases, namely Na^+^/K^+^-ATPase, Ca^2+^-ATPase, and Mg^2+^/Ca^2+^-ATPase, to elucidate whether these enzymes are a possible target site for these EOs. Furthermore, our research aims to gain a comprehensive understanding of the biochemical repercussions of these EOs on *A. ipsilon* with a specific focus on the modulation of detoxifying enzyme activity, namely α-esterase and GST. Additionally, we performed a molecular docking analysis to recognize the amino acids’ interactions, the lengths of hydrogen bonds (Å), the affinity (Kcal/mol), and the docking energy score of the major constituents of *O. majorana* and *R. officinalis* Eos, i.e., terpinene-4-ol and α-pinene, against the active site of α-esterase enzyme, compared to the co-crystallized ligand of this enzyme, DPF (diethyl hydrogen phosphate). 

## 2. Materials and Methods

### 2.1. Insect

A strain of *Agrotis ipsilon* was reared in the laboratory (26 ± 1 °C, 65 ± 5% RH, 16:8 [L:D] h) [1], away from any insecticide exposure. The newly hatched larvae were placed in a clean glass jar (1 L) covered with muslin and secured with a rubber band. They were daily fed fresh castor bean (*Ricinus communis* L.) leaves until they reached the third larval instar [50]. To avoid excessive cannibalism, the larvae were individually placed in small plastic cups (7.0 cm in diameter, 3.5 cm in height) with fresh castor bean leaves at the beginning of the fourth instar [50]. The pupae were kept surrounded with paper towels in glass jars until they matured. The adult moths were transferred to larger jars supplied with hung pieces of cotton moistened with 10% sugar solution [51] and covered with black muslin strips for egg deposition [6]. The eggs were collected daily and transferred to new jars, and the neonates were fed castor bean leaves as described above.

### 2.2. Chemicals

The following chemicals were obtained from Sigma-Aldrich (Sigma-Aldrich, St. Louis, MO, USA): Adenosine triphosphate (ATP), ouabain, trichloroacetic acid (TCA), L-glutathione reduced (GSH), 1-chloro-2,4 dinitrobenzene (CDNB), α-naphthyl acetate, and α-naphthol. All chemicals were of the highest grade.

### 2.3. Essential Oils Preparation and GC-Mass Analysis

The EOs of *Origanum majorana* and *Rosmarinus officinalis* were extracted from fresh leaves as described by [13]. The chemical composition of the EOs was identified using a GC Ultra-ISQ mass spectrometer (Thermo Scientific, Austin, TX, USA) (Table S1 in the Supplementary File).

### 2.4. Insect Susceptibility Assay

The EOs of *O. majorana* and *R. officinalis* were examined for their bioactivity on the 2nd instar larvae (24 h old) of *A. ipsilon*. The castor bean leaf dipping method was used with five concentrations of the EOs: 0.5, 1.0, 2.0, 4.0, and 8.0 mg mL^−1^ [52]. Three replicates (10 larvae/replicate) were prepared for each concentration. Water stock solutions of EOs were mixed in cups with Tween-20 (0.05%), as an emulsifying agent. In the control cups, the EO was replaced with water. The experiment was kept under the same insect-rearing conditions. After 24 h of larval feeding, the live larvae were transferred to clean jars and the treated castor bean leaves were replaced with untreated ones. Mortalities of larvae were recorded at 24, 48, 72, and 96 h post-treatment. The sublethal and lethal concentrations (LC_10_, LC_20_, LC_30_, and LC_70_) of the EOs at 96 h post-treatment were calculated using probit analysis [53].

### 2.5. Biochemical Assays

#### 2.5.1. ATPase Assays

##### Preparation of Insect’s Homogenate 

One thousand five hundred 2nd instar larvae of *A. ipsilon* in triplicate were treated with the LC_20_ (0.25 and 1.85 mg mL^−1^) and LC_70_ (0.49 and 4.41 mg mL^−1^) of *O. majorana* and *Rosmarinus officinalis*, respectively. Additionally, there were more than two hundred untreated larvae that were allowed to feed on untreated castor bean leaves. Forty-five live larvae were collected every 24, 48, 72, and 96 h post-treatment to determine the activity of Na^+^/K^+^-, Mg^2+^/Ca^2+^-, and Ca^2+^-ATPases in triplicate for each assay. They were weighed, rinsed, and homogenized in 10 mM Tris-HCl buffer (pH 7) containing 1 mM EDTA and 0.32 M Sucrose. The homogenate was then centrifuged at 4 °C at 2500× *g* for 10 min and the supernatant was centrifuged again at 22,000× *g* for 30 min. The supernatant was discarded, and the sediments were resuspended in Tris-HCl buffer (pH 7.4) containing 1 mM EDTA [29,54]. The samples were kept frozen at −20 °C until used for ATPase assays. 

##### Na^+^/K^+^-ATPase

The activity of Na^+^/K^+^-ATPase was determined using three groups of reaction systems containing 50 mM Tris-HCl buffer (pH 7), 5 mM MgCl_2_, and sample enzyme source according to the method described by [29]. Reaction system 1 contained 150 mM NaCl, and 20 mM KCl; reaction system 2 contained 1 mM ouabain in 50 mM Tris-HCl buffer (pH 7); and reaction system 3 was considered as control (contained 50 mM Tris-HCl buffer (pH 7), 5 mM MgCl_2_, and sample enzyme source). These reaction systems were repeated three times (1 mL per system). All reaction systems were incubated for 5 min at 37 °C, and then 1.5 mM of adenosine triphosphate (ATP) was added. Reaction systems were incubated again for 15 min at 37 °C before reaction systems 1 and 2 were terminated by adding 15% of ice-cold trichloroacetic acid (TCA). Reaction systems were mixed by inversion, and then the phosphorous stain (1% ammonium molybdate tetrahydrate in 0.5 N sulfuric acid) and 1% freshly prepared ascorbic acid were added. The absorbance was read at 625 nm (Jenway-7205UV/Vis Spectrophotometer) after the systems were incubated for 30 min at 25 °C.

##### Mg^2+^/Ca^2+^- and Ca^2+^-ATPases

Mg^2+^/Ca^2+^-ATPase and Ca^2+^-ATPase activities were assayed using two groups of reaction systems according to the method of [55] with some modifications [28]. In the Mg^2+^/Ca^2+^-ATPase assay determination, the two reaction systems (1 mL per system, with three replicates) contained 50 mM Tris-HCl buffer (pH 7), 1 mM MgCl_2_, 0.1 mM CaCl_2_, 10 mM KCl, 1 mM ouabain, and sample enzyme source. In the Ca^2+^-ATPase assay determination, the two reaction systems (1 mL per system, with three replicates) contained 50 mM Tris-HCl buffer (pH 7.4), 5 mM CaCl_2_, and 1 mM ouabain. Reaction system 1 in both determinations contained 1.5 mM ATP substrate while reaction system 2 contained 15% ice-cold trichloroacetic acid and 1.5 mM ATP substrate. Before adding the ATP substrate in both determinations, the two reaction systems were placed in a dry bath incubator at 37 °C. Reaction system 1 in both determinations was terminated by adding 15% of ice-cold trichloroacetic acid after 5 min. The phosphorous stain was added to the two reaction systems in both determinations before reading the absorbance at 625 nm.

ATPases were determined as described by [56,57] and the absorbance level was compared to a standard absorbance curve for known inorganic phosphate concentrations.

#### 2.5.2. Detoxification Enzymes Assay

##### α-Esterase Activity Assay

Three hundred 2nd instar larvae of *A. ipsilon* in triplicate were treated with the LC_10_ (131 and 399 mg/L) and LC_30_ (242 and 818 mg/L) of *O. majorana* and *Rosmarinus officinalis*, respectively. At 24, 48, 72, and 96 h, fifteen live larvae of the treatment or control groups were weighed, rinsed with distilled water, and homogenized in 40 mM potassium phosphate buffer containing 1 mM EDTA at pH 7. The homogenates were then centrifuged for 10 min at 14,000× *g*. The activity of α-esterase was determined in the supernatants according to [58] with small modifications [59]. The absorbance levels were compared to a standard curve of absorbance obtained from known concentrations of α-naphthol (50 mM methanolic stock solution). The specific activities of α-esterase were reported as μ moles of α-naphthol formed min^−1^ mg^−1^ protein.

##### Glutathione-S-Transferase Activity Assay

The GST enzyme activity was assayed using 1-chloro-2,4 dinitrobenzene (CDNB) as a substrate, based on the method of [60]. Three hundred 2nd instar larvae of *A. ipsilon* in triplicate were treated with the LC_10_ (131 and 399 mg/L) and LC_30_ (242 and 818 mg/L) of *O. majorana* and *R. officinalis*, respectively. At 24, 48, 72, and 96 h, fifteen live larvae of the treatment or control groups were weighed, rinsed with distilled water, and homogenized in 100 mM potassium phosphate buffer containing 1 mM EDTA at pH 6.5. The homogenates were then centrifuged at 10,000× *g* for 10 min. The GST activity was determined in the supernatant as described by [60]. The GST-specific activities were expressed as nmols min^−1^ mg^−1^ protein. Three replicates from the treated and untreated (control) groups were used for all enzyme assays and correction for non-enzymatic conjugation in the samples was made.

#### 2.5.3. Protein Assay

Protein concentration (mg protein/mL homogenates) was determined according to [61] and was used for standardization.

### 2.6. Molecular Docking

The structure of terpinene-4-ol and α-pinene, the major constituents of *O. majorana* and *Rosmarinus officinalis*, respectively, were created in the PDB file format using the Gaussian 09 software outputs. α-esterase crystal structure (PDB ID: 4FNM) was downloaded from the protein data bank (http://www.rcsb.org/,accessed on 26 June 2024). The molecular docking studies were performed using the MOE 2015 software. The co-crystallized ligand was re-docked in its original enzyme structure using the default parameters. 

### 2.7. Statistical Analysis

The data underwent assessment to ensure that they met the assumptions required for parametric tests. Continuous variables were evaluated for normality using both the Shapiro–Wilk and Kolmogorov–Smirnov tests. The lethal and sublethal concentrations of the EOs were estimated by probit analysis [53] utilizing the Log Dose Probit line^®^ software (https://www.ehabsoft.com/ldpline/, accessed on 26 June 2024). Effects of EO treatments, and time post-treatment on the ATPase and detoxification enzymes (α-esterase, and GST) were subjected to ANOVA (Type II) using the generalized linear method (GLM) procedure, followed by Tukey’s multiple comparisons test to compare the significance level between every two groups (*p* ≤ 0.05). This analysis and data representation as figures were conducted using GraphPad Prism, version 9.3.1 (GraphPad Software LLC, San Diego, CA, USA). The EO treatments and times post-treatment were considered fixed factors while the enzyme activity was treated as a random factor. Within the same day, the means of enzyme activities were separated using Duncan’s multiple range test (*p* ≤ 0.05) (SPSS, V. 19.0, IBM Corporation, New York, NY, USA). 

## 3. Results

### 3.1. Chemical Composition of Origanum majorana and Rosmarinus officinalis Essential Oils 

**Figure 1:**
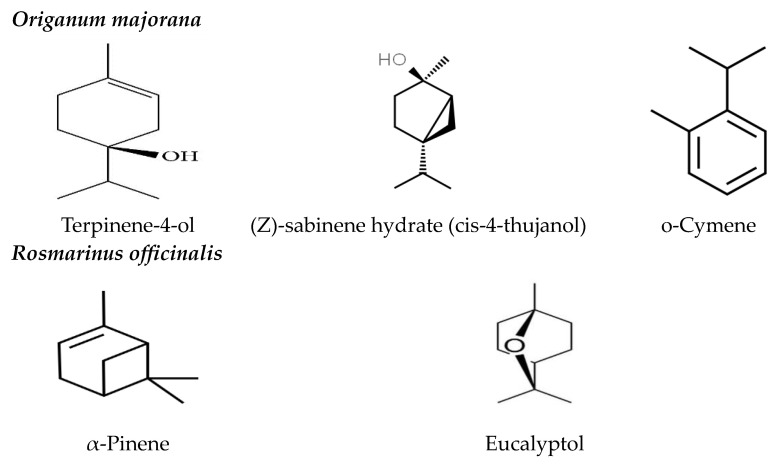
Chemical structure of the main bioactive compounds of *Origanum majorana* and *Rosmarinus officinalis* EOs.

Our results indicated that the major components of *O. majorana* EO were terpinene-4-ol (39.35%), (z)-sabinene hydrate (cis-4-thujanol) (18.40%), and o-cymene (11.72%), and the major constituents of *R. officinalis* EO were α-pinene (34.29%) and eucalyptol (29.70%) (Figure 1, and Tables S1 and S2 in the Supplementary File).

### 3.2. Toxicity of the Tested EOs to A. ipsilon Larvae

The insecticidal activity of *O. majorana* and *R. officinalis* EOs on *A. ipsilon* larvae was evaluated using the leaf dipping technique and data are shown in Table 1. The LC_10_, LC_20,_ LC_30_, and LC_70_ (mg mL^−1^) were 0.13, 0.25, 0.39, and 1.85 mg mL^−1^ for *O. majorana* and 0.24, 0.49, 0.82, and 4.41 mg mL^−1^ for *R. officinalis*, respectively.

### 3.3. Biochemical Assays 

#### 3.3.1. ATPase Assays

**Figure 2:**
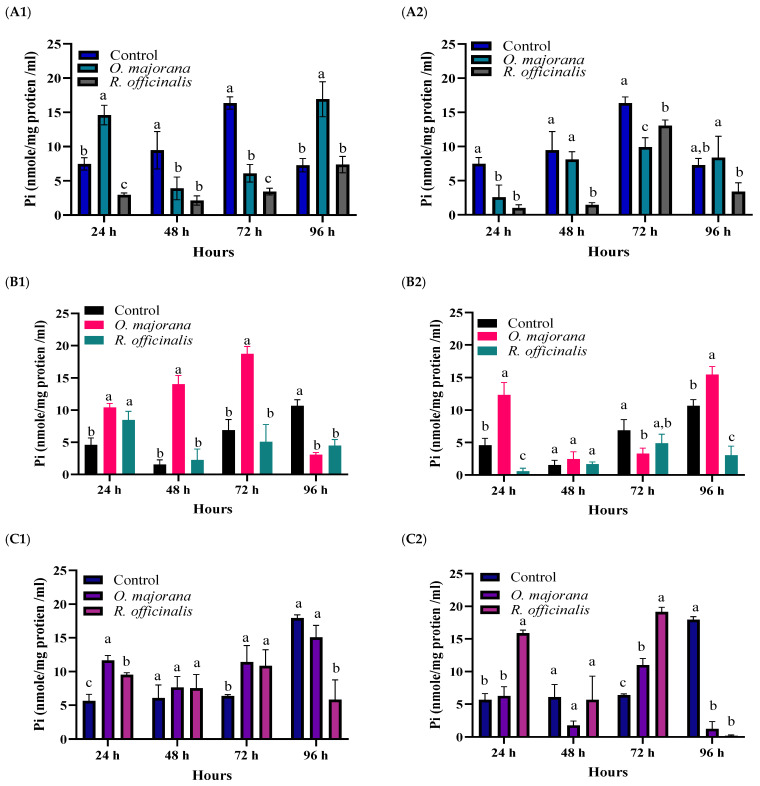
The specific activity of (**A**) Na^+^/K^+^-ATPases at 1: LC_20_, 2: LC_70_, (**B**) Mg^2+^/Ca^2+^-ATPases at 1: LC_20_, 2: LC_70_, and (**C**) Ca^2+^-ATPases at 1: LC_20_, 2: LC_70_ of *Origanum majorana* and *Rosmarinus officinalis* EOs compared to the control group in *A. ipsilon* larvae at 24, 48, 72, and 96 h post-treatment. For each interval, the columns with different letters are significantly different (*p* ≥ 0.05).

Effects of the EOs at LC_20_ and LC_70_ at 24, 48, 72, and 96 h post-treatment on the Na^+^/K^+^-, Mg^2+^/Ca^2+^-, and Ca^2+^-ATPases of the *A. ipsilon* 2nd instar larvae were studied (Figure 2). Two-way ANOVA (GLM) and Tukey’s multiple comparisons test were utilized to compare the treatments with the control. Additionally, both tested EOs were compared with each other at different time intervals (Table 2).

##### Na^+^/K^+^-ATPase 

At LC_20_, a significant difference was found among treatments (F (2,6) = 290.4, *p* ≤ 0.05) at different time intervals (F (3,8) = 17.02, *p* ≤ 0.05), and among the interaction between treatments and time (F (6,18) = 28.14, *p* ≤ 0.05) in the specific activity of Na^+^/K^+^-ATPases. Similar results were found for LC_70_ of the same EO, and the corresponding values were (F (2,6) = 41.83, *p* ≤ 0.05) for treatments, (F (3,18) = 58.35, *p* ≤ 0.05) for time, and (F (6,18) = 7.030, *p* ≤ 0.05) for the interaction between treatments and time. On the other hand, at LC_20_ Tukey’s multiple comparisons displayed a significant (*p* ≤ 0.05) difference between the control and *O. majorana*, the control and *R. officinalis*, and *O. majorana* versus *R. officinalis* at all time intervals, except for the control and *R. officinalis* at 96 h post-treatment. Similarly, at LC_70_ of both EOs, Tukey’s multiple comparisons indicated a significant difference (*p* ≤ 0.05) between the control and *O. majorana*, the control and *R. officinalis*, and *O. majorana* versus *R. officinalis* at all time intervals, except for the control and *R. officinalis* at 24 and 72 h post-treatment.

Overall, *O. majorana* showed a contrasting effect to Na^+^/K^+^-ATPase, as the LC_20_ significantly inhibited the Na+-K+ pump activity at 48 and 72 h after-treatment although it activated it at 24 and 96 h after-treatment. In addition, the LC_70_ of the same EO inhibited this activity at 24 and 72 h post-treatment but activated it at 24 h post-treatment. However, *R. officinalis* suppressed the Na^+^/K^+^ pump across all time intervals.

##### Mg^2+^/Ca^2+^-ATPases

At LC_20_, discernible variations were observed in the effects of the EOs on Mg^2+^/Ca^2+^-ATPases across treatments. These distinctions were evident in treatment (F (2,6) = 75.07, *p* ≤ 0.05), time (F (2,8) = 20.45, *p* ≤ 0.05), and interaction between treatment and time (F (6,18) = 44.97, *p* ≤ 0.05). Similarly, at LC_70_ of the same EOs, the corresponding values were (F (2,6) = 68.27, *p* ≤ 0.05) for treatment, (F (2,8) = 70.03, *p* ≤ 0.05) for time, and (F (6,18) = 33.02, *p* ≤ 0.05) for the interaction between treatments and time. 

On the other hand, at LC_20_, Tukey’s test displayed a significant difference (*p* ≤ 0.05) between the control and *O. majorana* at all time intervals. Additionally, distinctions were noted between the control and *R. officinalis* at 24 and 96 h, as well as between *O. majorana* and *R. officinalis* at 48 and 72 h. Similarly, at LC_70_ of both EOs, Tukey’s test revealed significant (*p* ≤ 0.05) differences between the control and *O. majorana* at 24, 72, and 96 h. Moreover, distinctions were observed between the control and *R. officinalis* at 24 and 96 h, and between *O. majorana* and *R. officinalis* at 24 and 72 h post-treatment.

Overall, similar to the Na+-K+ pump, *O. majorana* exhibited the same trend between activation and inhibition of Mg^2+/^Ca^2+^-ATPase at different time intervals in response to LC_20_ and LC_70_. However, both tested concentrations of *R. officinalis*, LC_20_ and LC_70_, significantly inhibited the activity of Ca^2+^ Mg^2+^-ATPase at 96 h post-treatment.

##### Ca^2+^-ATPases

At LC_20_, notable distinctions were observed in the impact of EOs on Ca^2+^-ATPases across various treatments. These distinctions were evident in treatment (F (2,6) = 12.24, *p* ≤ 0.05), time (F (2,8) = 17.34, *p* ≤ 0.05), and the interaction between treatments and time (F (6,18) = 15.20, *p* ≤ 0.05). Similarly, at LC_70_ of the same EOs, the corresponding values were (F (2,6) = 62.49, *p* ≤ 0.05) for treatment, (F (2,8) = 47.45, *p* ≤ 0.05) for time, and (F (6,18) = 70.72, *p* ≤ 0.05 for the interaction between treatments and time.

On the other hand, at LC_20_, Tukey’s test displayed a significant (*p* ≤ 0.05) difference between the control and *O. majorana* at 24 and 72 h, and between the control and *R. officinalis* at 24, 72, and 96 h. Additionally, a distinction was observed between *O. majorana* and *R. officinalis* at 96 h. Similarly, at LC_70_ of both EOs, Tukey’s test uncovered significant (*p* ≤ 0.05) differences between the control and *O. majorana* at 48, 72, and 96 h, the control and *R. officinalis* at 24, 72, and 96 h, and *O. majorana* and *R. officinalis* at 24, 48, and 72 h post-treatment.

Overall, the *Ca^2+^-ATPases* were significantly inhibited at 96 h post-treatment with LC_70_ of both *O. majorana* and *R. officinalis* and with LC_20_ of *R. officinalis* only.

#### 3.3.2. Detoxification Enzymes Assay

**Figure 3:**
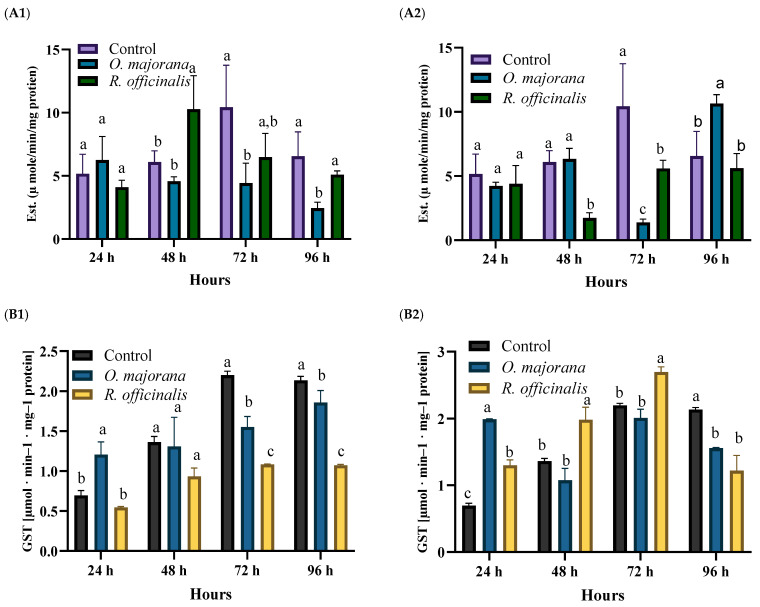
The specific activity of (**A**) α-esterase (Est.) at 1: LC_10_, 2: LC_30_, and (**B**) glutathione-S-transferase (GST) at 1: LC_10_, 2: LC_30_ concentrations *Origanum majorana* and *Rosmarinus officinalis* EOs compared to control group (untreated) on *A. ipsilon* larvae, at 24, 48, 72 and 96 h post-treatment. For each time point, columns with different letters are significantly different (*p* ≥ 0.05).

The effects of LC_20_ and LC_70_ of the EOs on carboxylesterase and GST activities in the *A. ipsilon* 2nd instar larvae at different time intervals (24, 48, 72, and 96 h) post-treatments are presented in Figure 3. Two-way ANOVA and Tukey’s test were used to compare the treatments with the control. Additionally, both tested EOs were compared with each other at different time intervals (Table 3).

##### α-Esterase

The LC_10_ of *O. majorana* EO significantly inhibited the α-esterase activity at 96 h post-treatment. Additionally, the LC_30_ of the same EO significantly increased the activity of α-esterase at 96 h post-treatment, while it inhibited the activity of α-esterase at 72 h post-treatment.

The LC_10_ of *R. officinalis* significantly increased the activity of α-esterase at 48 h post-treatment. In addition, the LC_30_ of the same EO significantly inhibited the activity of α-esterase at 48, 72, and 96 h post-treatment.

##### Glutathione-S-Transferase (GST)

Overall, the LC_10_ of *O. majorana* EO significantly increased the GST activity at 24 h post-treatment but inhibited the activity of GST at 72 and 96 h post-treatment. Additionally, the LC_30_ of the same EO significantly increased the activity of GST at 24 h post-treatment, while it significantly inhibited the activity of GST at 96 h post-treatment.

The LC_10_ of *R. officinalis* significantly inhibited the activity of GST at 72 and 96 h post-treatment. In addition, the LC_30_ of the same EO significantly increased the activity of GST at 48 and 72 h post-treatment but inhibited the activity of GST at 96 h post-treatment.

### 3.4. Molecular Docking 

Molecular docking was performed for terpinene-4-ol and α-pinene, the major constituents of *O. majorana* and *R. officinalis* EOs, respectively, against the active site of α-esterase enzyme (PDB ID: 4FNM). The binding and interactions with the significant amino acids were carried out by docking studies. The docking process was validated using the co-crystallized ligand DPF (diethyl hydrogen phosphate). Interactions of amino acids, lengths of hydrogen bonds (A°), affinity (Kcal/mol), and docking energy score are shown in Table 4. 

**Figure 4:**
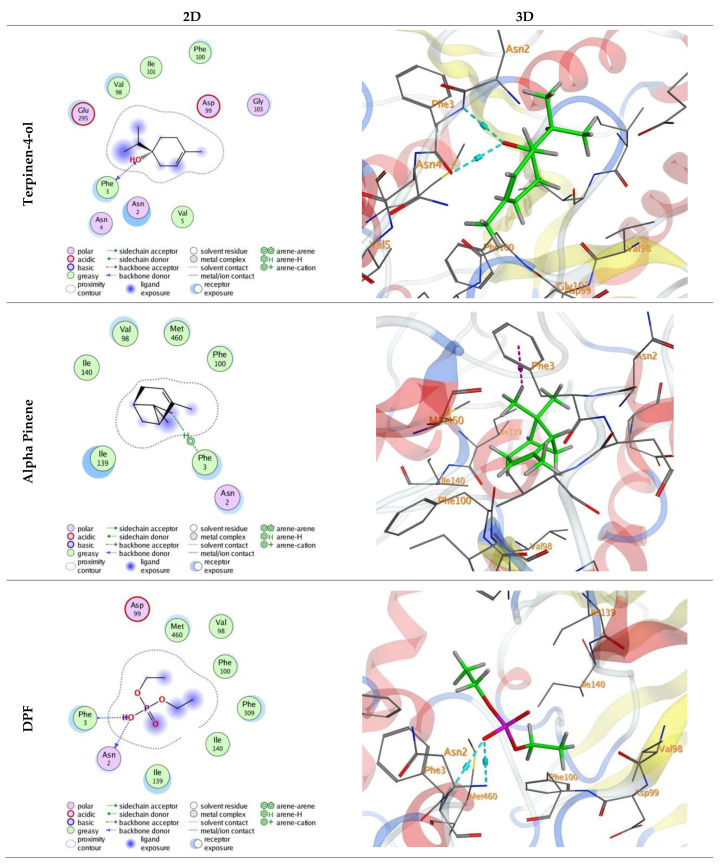
Diagrams of 2D and 3D interactions of the major bioactive components of *Origanum majorana* (terpinene-4-ol) and *Rosmarinus officinalis* (α-pinene) and diethyl hydrogen phosphate (DPF) in the active site of α-esterase (PDB ID: 4FNM). Hydrogen bonds are displayed in cyan and H-pi-bonds in magenta.

Terpinene-4-ol and α-pinene gave energy scores (S) of −4.51 and −4.29 Kcal/mol, respectively. The binding pattern of terpinene-4-ol showed two hydrogen bonds with the amino acid residue PHE 3 (Figure 4). The interaction between α-pinene and α-esterase protein was stabilized through one H-arene contact with PHE 3 (Figure 4). Finally, DPF had an energy score (S) of −4.67 kcal/mol and produced two H-bonds with ASN 2 and PHE 3 amino acids (Figure 4). 

## 4. Discussion

Egypt’s fertile agricultural terrain provides an ideal environment for cultivating aromatic plants like rosemary and marjoram [62]. These perennials offer economic benefits as they regrow after harvest, thereby eliminating the need for labor-intensive annual replanting [63]. Egypt’s climate further supports its growth across diverse regions [64,65]. Leveraging the plentiful availability of these plants in Egypt to produce these EOs and their application in pest management aligns with the principles of sustainable agriculture, providing a safe and effective solution.

Furthermore, the EOs derived from these plants are multifunctional as they possess medicinal properties [66,67] and are used as flavorings [68,69]. In comparison to chemical insecticides, these EOs present a safer alternative. Our research highlights the potent insecticidal properties of marjoram and rosemary EOs against *Agrotis ipsilon* larvae, a notably invasive and harmful pest.

The GC/MS analysis of the tested EOs revealed that the major constituents of *O. majorana* EO are terpinen-4-ol (39.35), sabinene hydrate (cis-4-thujanol) (18.40), and o-cymene (11.72). These findings align with numerous studies that have identified these constituents as the primary components of *O. majorana* EO [33,40,70]. 

Similarly, the analysis showed that the hydrocarbon monoterpene, α-pinene (34.29), and the oxygenated monoterpene, eucalyptol (1,8-cineole) (29.70), are the main components of *R. officinalis* EO. These findings align with many studies that have reported eucalyptol (1,8-cineole) and α-pinene as the major constituents of *R. officinalis* EO [71,72]. However, the compositional profile of *R. officinalis* EO exhibits significant variability, with geographical origin emerging as a key influencing factor [33]. The three primary constituents, 1,8-cineole (eucalyptol), camphor, and α-pinene, associate the observed chemotype of rosemary EO profile with the Mediterranean coastal region [73].

Furthermore, several studies have demonstrated the pesticide action of eucalyptol, which is identified in various EOs [74], on different insect species such as *Tenebrio molitor* (Coleoptera: Tenebrionidae) [70], *Musca domestica* (Diptera: Muscidae) [75], *Rhyzopertha dominica* (Coleoptera: Bostrychidae), *Callosobruchus maculatus* (Coleoptera: Bruchidae), *Sitophilus oryzae* (Coleoptera: Curculionidae) [76], and *Acanthoscelides obtectus* (Coleoptera: Bruchidae) [39]. Moreover, o-cymene from *O. majorana* and eucalyptol from *R. officinalis* EOs have shown various levels of toxicity and repellence against *Liposcelis bostrychophila* and *Tribolium castaneum* [77].

The potential insecticidal activity of the EOs from *O. majorana* (marjoram) and *R. officinalis* (rosemary) against various species has been investigated by a plethora of studies and pilot tests [31,78,79,80]. Our research corroborated these findings, demonstrating significant insecticidal activity of these EOs against *A. ipsilon* larvae. Notably, while *O. majorana* oil exhibited higher toxicity than *R. officinalis* against the 2nd instar larvae of *A. ipsilon*, the latter showed considerable toxicity, compared to the control.

The existence or absence of the phosphate group distinguishes four types of ATPases: P, V, F, and ABC ATPases [81]. The P-type is found in all living cells, regulating the transport of ions across membranes. It transports ions like protons, sodium, potassium, calcium, and heavy metals across diverse biological membrane systems [82]. Different types of P-type ATPases were found to be the target site of insect management tools like pesticides, i.e., chlorinated hydrocarbons and pyrethroids [83,84,85,86] or their bio-alternative, i.e., plant EOs [87,88]. 

In the current study, we determined the specific activity of three types of P-type ATPases, Na^+^/K^+^-ATPase, Mg^2+^/Ca^2+^-ATPase, and Ca^2+^-ATPase, at different time intervals post-treatment with lethal and sub-lethal concentrations of *O. majorana* and *R. officinalis*. This was based on the hypothesis that these ATPases might affect the tested EO toxicity to *A. ipsilon* larvae. Surprisingly, significant effects of these ATPases were observed for different concentrations of the tested EOs. Additionally, an interaction between time and treatment was also recorded. 

*O. majorana* showed a contrasting effect on the Na^+^/K^+^-ATPase or Na^+^-K^+^ pumps. This contrasting effect between activation and inhibition might indicate that the Na^+^-K^+^ pump is not the main target site of this EO. On the contrary, *R. officinalis* inhibited the Na^+^-K^+^ pump activity at both tested concentrations, LC_20_ and LC_70_, at almost all-time intervals post-treatment, except for at 96 h post-treatment of the LC_20_. 

The Na^+^-K^+^ pump exists in the plasma membrane of almost all animal cells, and functions as an antiporter, actively pumping Na^+^ out of the cell and pumping K^+^ in, thereby maintaining the cell’s equilibrium and the cell’s ability to generate electrical impulses [89,90]. The main difference between this pump and voltage-gated sodium channels (VGSCs) lies in their function and mechanism of action. The former is an active pump that uses energy to maintain ion gradients, while the latter are passive channels that respond to changes in membrane potential to generate action potentials. Their roles in the action of pyrethroids also differ, with the VGSCs being the primary target of these insecticides [91]. 

The suppression of this pump observed in the current study following exposure to *R. officinalis* across all time intervals suggests that this pump is a potential target for this EO. This suppression might elevate the concentration of Na^+^ ions, triggering heightened neuronal excitation, and ultimately causing a knockdown effect and death. In harmony with this, a dose–response and time-course study conducted by [92] reported that the Na^+^-K^+^ pump inhibition is related to the knockdown effect of the decalesides, a novel category of bio-insecticides. However, an electrophysiology study, using the patch clamp technique, is needed to confirm this hypothesized mode of action of *R. officinalis*.

The Mg^2+^/Ca^2+^-ATPase, which shows the same mechanism as the Na^+^/K^+^-ATPase, can pump calcium ions against a concentration gradient. The calcium gradients made by this enzyme are critical to muscle relaxation [93]. In the current work, *O. majorana* showed the same trend, as shown in the Na^+^-K^+^ pump, between activation and inhibition of Mg^2+^/Ca^2+^-ATPase at different time intervals in response to LC_20_ and LC_70_. The LC_20_ of this EO inhibited the activity of the Mg^2+^/Ca^2+^-ATPase at 96 h post-treatment. This might indicate that the Mg^2+^/Ca^2+^-ATPase target site is one of the target sites of *O. majorana* but not the major one. 

On the contrary, both tested concentrations of *R. officinalis*, LC_20_ and LC_70_, significantly inhibited the activity of Mg^2+^/Ca^2+^-ATPase at 96 h post-treatment, the time at which we recorded the highest mortality rate in *A. ipsilon* larvae in the concentration gradient bioassay. This inhibition of the Mg^2+^/Ca^2+^-ATPase leads to inhibition in muscle relaxation, which might be a response to the long-lasting inhibition in the Na^+^-K^+^ pump due to treatment with the two tested concentrations of *R. officinalis* recorded in the current work. 

Ca^2+^-ATPase, or the Ca^2+^ pump, exists in the sarcoplasmic reticulum membrane of skeletal muscle cells and is responsible for about 90% of the organelle membrane protein. This pump serves as an intracellular store of Ca^2+^ and accounts for moving Ca^2+^ from the cytosol to the sarcoplasmic reticulum [24]. As reported by [94], the regulation of Ca^2+^ inner or outer nerve membranes is chiefly performed by Mg^2+^/Ca^2+^-ATPase and Ca^2+^-ATPase. In the current work, the Ca^2+^ pump was significantly inhibited at 96 h post-treatment with LC_70_ of both *O. majorana* and *R. officinalis* and with LC_20_ of *R. officinalis* only. This finding supports our hypothesis that Mg^2+^/Ca^2+^-ATPase and Ca^2+^-ATPase are target sites for *O. marjoram* and might be a major target for *R. officinalis.*

The activities of α-esterase, mixed-function oxidase, and Glutathione S-transferase (GST) have been the subject of extensive research, particularly on insects’ exposure to xenobiotics [95]. These detoxification enzymes serve as potent biological indicators due to their sensitivity in signaling exposure to chemicals [96]. However, their activity after xenobiotic exposure has been reported to show a varied level of activity between activation and inhibition in different insects after exposure to various chemicals [28]. Hence, the activity of these detoxifying enzymes does not directly reveal the target site of a compound. Instead, it provides insights into how the insect’s enzymatic system interacts with these xenobiotic substances, essentially shedding light on the compound’s mode of action. In our research, we assessed the α-esterase and GST activities as an indicator of the detoxification process of the EOs in *A. ipsilon* larvae. While cytochrome P450s are undoubtedly important in detoxification processes, our previous findings [13] suggested that GST and esterases may play a more direct role in mediating the effects of our tested EOs. 

The data revealed that the two tested EOs, *O. majorana* and *R. officinalis*, exhibited a varied level of activation and inhibition of the detoxification enzymes, α-esterase and GST, over time post-treatment. The increased activity of detoxification enzymes following xenobiotic treatment may represent a direct adaptive response aimed at neutralizing toxic compounds and detoxifying insecticides, thereby enhancing survival rates [97]. Furthermore, Ref. [98] suggested that the induction of detoxification enzymes is associated with increased tolerance of insects to insecticides.

However, in the current work, the most common effect of both tested EOs on α-esterase and GST was inhibition. Insects utilize such detoxification enzymes as α-esterase and GST to metabolize the secondary metabolites of plants, thereby protecting themselves from oxidative damage [99]. This suggests that the mortality of *A. ipsilon* larvae following exposure to *R. officinalis* and *O. majorana* EOs might be a consequence of the reduced activity of α-esterase and GST. Our findings suggest that α-esterase and GST play a part in the detoxification process of *O. majorana* and *R. officinalis* in *A. ipsilon* larvae. However, further research, at the molecular level, could provide more precise insights. 

Our current results align with those of [100], who observed varied levels in the activity of AChE, carboxylesterase, and GST in the cereal weevil, *Sitophilus zeamais*, in response to *Melaleuca alternifolia* EO over time post-treatment. However, the varied level recorded in their study in the EO-treated insects was consistently lower than that recorded in the untreated insects at all time intervals, presenting a contrasting result with ours. This variation between our study and theirs could be attributed to the different concentrations used; they used a lethal concentration (LC_50_), while we used sublethal ones (LC_10_ and LC_30_). Additionally, they employed a fumigation method to treat their insects, whereas we used the leaf dipping technique. This latter point suggests a potential future comparative study testing the same EOs using different bioassay methods to identify the most efficient bioassay method for these EOs against *A. ipsilon* larvae.

Furthermore, the inhibition trend of detoxification enzymes by the tested EOs in the current work highlights the potential of using these EOs to enhance the toxicity of synthetic insecticides as previously suggested by [101]. In accordance with this suggestion, [102] reported that various plant EOs have been shown to inhibit detoxification enzymes such as GST and cytochrome P450 monooxygenases of *Aedes aegypti* and *Anopheles gambiae*, both pyrethroid-susceptible and pyrethroid-resistant strains, increasing the efficacy of pyrethroids against the resistant populations of these pests. Additionally, lemongrass EO was found to synergize the toxic effect of certain insecticides on *Bemisia tabaci* adults [103]. These findings suggest that EOs could be valuable synergistic agents in conventional insecticides. Future research should focus on exploring the precise mechanisms of enzyme inhibition by these EOs, synergistic effects in various pest management scenarios, and ensuring environmental safety. Large-scale field trials are also recommended to assess the practicality and long-term benefits of integrating EOs with traditional insecticides in pest control programs across urban, public health, and agricultural settings.

The molecular docking results indicated that terpinene-4-ol and α-pinene, the major constituents of *O. majorana* and *R. officinalis* EOs, respectively, exhibited favorable energy scores (S); −4.51 and −4.29 Kcal/mol, respectively, which closely resemble the score of the diethyl hydrogen phosphate (DPF) ligand (−4.67). This alignment with the toxicity assay data further supports the observation that *O. majorana* is more toxic than *R. officinalis* to *A. ipsilon* larvae. Additionally, the molecular docking results reinforce our earlier hypothesis, initially derived from the biochemical analysis of detoxification enzymes, regarding the involvement of the detoxification enzyme α-esterase in the response of *A. ipsilon* larvae to both tested EOs. 

## 5. Conclusions

This study indicates that the Na+-K+ pump may be a primary target for *Rosmarinus officinalis*. Additionally, the Mg^2+^/Ca^2+^-ATPase and Ca^2+^ pumps may also be targeted by this EO. However, while *Origanum marjoram* may influence these pumps, they might not be its primary targets. Furthermore, the findings regarding detoxification enzymes suggest that α-esterase and GST play roles in the detoxification process of these EOs in *A. ipsilon* larvae. Overall, *O. majorana* and *R. officinalis* EOs can be promising insecticides in organic farming and IPM programs for *A. ipsilon* management. Additionally, in silico studies could serve as a powerful tool to validate and enhance the understanding of the toxicity and biochemical data. To conclude, our research provided good insights; however, a more granular understanding of our results can be achieved through subsequent research employing advanced molecular and electrophysiological methods.

## Figures and Tables

**Table 1:** Larvicidal activity (in mg mL^−1^) of *Origanum majorana* and *Rosmarinus officinalis* essential oils (EOs) against *A*. *ipsilon* 2nd instar larvae at 96 h post-treatment.

EOs	No.	LC_10_ (mg mL^−1^) (95% CL)	LC_20_ (mg mL^−1^) (95% CL)	LC_30_ (mg mL^−1^) (95% CL)	LC_70_ (mg mL^−1^) (95% CL)	Slope (SE)	χ^2^
*O. majorana*	120	0.13 (0.02–0.29)	0.25 (0.05–0.46)	0.39 (0.13–0.64)	1.85 (1.30–3.25)	1.57 (0.37)	0.11
*R. officinalis*	120	0.24 (0.04–0.51)	0.49 (0.14–0.85)	0.82 (0.34–1.27)	4.41 (2.93–9.46)	1.43 (0.32)	0.11

LC_10_, LC_20_, LC_30_, and LC_70_ are the concentrations of EOs that cause mortality to 10, 20, 30, and 70% of the *A. ipsilon* 2nd instar larvae population, respectively.

**Table 2:** Two-way ANOVA (GLM procedure) and Tukey’s multiple comparisons test for the effects of LC_20_ and LC_70_ of *Origanum majorana* and *Rosmarinus officinalis* EOs at different time intervals post-treatments on the Na^+^/K^+^-, Mg^2+^/Ca^+^-, and Ca^2+^-ATPases of *A. ipsilon* 2nd instar larvae.

Two-Way ANOVA (GLM)					Tukey’s Multiple Comparisons Test (*p*-Value) ^3^			
Source of Variation	SS ^1^	MS ^2^	F (df)	*p*	Time ^4^	C. ^5^ vs. *O. majorana*	C. vs. *R. officinalis*	*O. majorana* vs. *R. officinalis*
Na^+^/K^+^-ATPase (LC_20_)								
Treatments (Tr)	317.2	158.6	290.4 (2,6)	<0.0001	**24**	**<0.0001**	**0.0023**	<0.0001
Time (Ti)	133.5	44.51	17.02 (3,18)	<0.0001	**48**	**0.0002**	**<0.0001**	0.3102
Ti × Tr	441.5	73.59	28.14 (6,18)	<0.0001	**72**	**<0.0001**	**<0.0001**	0.0812
					**96**	**<0.0001**	0.9968	<0.0001
Na^+^/K^+^-ATPase (LC_70_)								
Treatments (Tr)	176.3	88.17	41.83 (2,6)	=0.0003	**24**	**0.002**	**<0.0001**	0.446
Time (Ti)	437.6	145.9	58.35 (3,18)	<0.0001	**48**	0.5416	**<0.0001**	**<0.0001**
Ti × Tr	105.4	17.57	7.030 (6,18)	0.0006	**72**	**<0.0001**	**0.0386**	0.0526
					**96**	0.6676	**0.014**	**0.0017**
Mg^2+^/Ca^2+^-ATPase (LC_20_)								
Treatments (Tr)	298.7	149.3	75.07 (2,6)	<0.0001	**24**	**<0.0001**	**0.0051**	0.2008
Time (Ti)	108.2	36.05	20.45 (3,18)	<0.0001	**48**	**<0.0001**	0.8055	**<0.0001**
Ti × Tr	475.7	79.28	44.97 (6,18)	<0.0001	**72**	**<0.0001**	0.2486	**<0.0001**
					**96**	**<0.0001**	**<0.0001**	0.4246
Mg^2+^/Ca^2+^-ATPase (LC_70_)								
Treatments (Tr)	206.5	103.2	68.27 (2,6)	<0.0001	**24**	**<0.0001**	**0.0009**	**<0.0001**
Time (Ti)	280.5	93.51	70.03 (3,18)	<0.0001	**48**	0.6218	0.9889	0.709
Ti × Tr	264.5	44.09	33.02 (6,18)	<0.0001	**72**	**0.0029**	0.1164	0.2417
					**96**	**0.0001**	**<0.0001**	**<0.0001**
Ca^2+^-ATPase (LC_20_)								
Treatments (Tr)	61.56	30.78	12.24 (2,6)	0.0076	**24**	**0.0007**	**0.028**	0.3018
Time (Ti)	162.0	54.00	17.34 (3,18)	<0.0001	**48**	0.5091	0.5648	0.9953
Ti × Tr	284.2	47.36	15.20 (6,18)	<0.0001	**72**	**0.0041**	**0.011**	0.9118
					**96**	0.1245	**<0.0001**	**<0.0001**
Ca^2+^-ATPase (LC_70_)								
Treatments (Tr)	175.4	87.69	62.49 (2,6)	<0.0001	**24**	0.8518	**<0.0001**	**<0.0001**
Time (Ti)	302.7	100.9	47.45 (3,18)	<0.0001	**48**	**0.0023**	0.9355	**0.0055**
Ti × Tr	902.2	150.4	70.72 (6,18)	<0.0001	**72**	**0.0014**	**<0.0001**	**<0.0001**
					**96**	**<0.0001**	**<0.0001**	0.6338

^1^ SS = sums of squares, ^2^ MS = mean source/square, ^3^ *p*-values below 0.05 are displayed in bold. ^4^ Times post-treatment, ^5^ Control group (untreated).

**Table 3:** Two-way ANOVA (GLM procedure) and Tukey’s multiple comparisons test for the effects of LC_10_ and LC_30_ of *Origanum majorana* and *Rosmarinus officinalis* on the detoxification enzymes (α-esterase (Est.) and GST) of *A. ipsilon* 2nd instar larvae at different time intervals post-treatment.

Two-Way ANOVA (GLM)					Tukey’s Multiple Comparisons Test (*p* Value) ^3^			
Source of Variation	SS ^1^	MS ^2^	F (df)	*p*	Time ^4^	C. ^5^ vs. *O. majorana*	C. vs. *R. officinalis*	*O. majorana* vs. *R. officinalis*
Est. (LC_10_)								
Treatments (Tr)	46.2	23.1	5.44 (2,6)	**0.0449**	**24**	0.7201	0.7315	0.2924
Time (Ti)	41.2	13.7	5.51 (3,18)	**0.0073**	**48**	0.5264	**0.0167**	**0.0012**
Ti × Tr	94.9	15.8	6.35 (6,18)	**0.0010**	**72**	**0.0007**	**0.0246**	0.3264
					**96**	**0.0188**	0.5538	0.1622
Est. (LC_30_)								
Treatments (Tr)	44.7	22.3	5.05 (2,6)	0.0518	**24**	0.6926	0.7744	0.9895
Time (Ti)	52.3	17.4	15.9 (3,18)	**<0.0001**	**48**	0.9756	**0.0022**	**0.0013**
Ti × Tr	163	27.2	24.7 (6,18)	**<0.0001**	**72**	**<0.0001**	**0.0008**	**0.003**
					**96**	**0.0039**	0.689	**0.0005**
GST (LC_10_)								
Treatments (Tr)	3.27	1.64	75.5 (2,6)	**<0.0001**	**24**	**0.0003**	0.3831	**<0.0001**
Time (Ti)	4.40	1.47	83.8 (3,18)	**<0.0001**	**48**	0.8816	**0.0021**	**0.0066**
Ti × Tr	1.49	0.25	14.1 (6,18)	**<0.0001**	**72**	**<0.0001**	**<0.0001**	**0.0008**
					**96**	0.0511	**<0.0001**	**<0.0001**
GST (LC_30_)								
Treatments (Tr)	0.26	0.13	2.04 (2,6)	0.2448	**24**	**<0.0001**	**0.0009**	**0.0003**
Time (Ti)	5.00	1.67	33.0 (3,18)	**0.0004**	**48**	0.0871	**0.0007**	**<0.0001**
Ti × Tr	5.60	0.93	41.7 (6,18)	**<0.0001**	**72**	0.3013	**0.0039**	**0.0003**
					**96**	**0.0013**	**<0.0001**	**0.0407**

^1^ SS = sums of squares, ^2^ MS = mean source/square, ^3^ *p*-values below 0.05 are displayed in bold. ^4^ Time post EO treatment, ^5^ Control group (untreated).

**Table 4:** Docking interaction of terpinen-4-ol, α-pinene, and DPF (diethyl hydrogen phosphate) inside α-esterase (PDB ID: 4FNM) active sites.

Compound	Energy Score (S) (Kcal/mol)	Affinity Bond Strength (Kcal/mol)	Affinity Bond Length (in A° from the Main Residue)	Amino Acids	Ligand	Interaction
**terpinene-4-ol**	−4.51	−0.9	3.19	PHE 3	O 14	H-donor
		−1.0	2.84	PHE 3	O 14	H-acceptor
**α-** **pinene**	−4.29	−0.6	4.57	PHE 3	C 23	H-pi
**DPF (diethyl hydrogen phosphate)**	−4.67	−1.0	3.29	ASN 2	O 3	H-donor
		−0.8	3.20	PHE 3	O 3	H-acceptor

## Data Availability

The datasets generated during and/or analyzed during the current study are available upon reasonable request from the corresponding author.

## Supplementary Materials

The following supporting information can be downloaded at: https://www.mdpi.com/article/10.3390/insects15070483/s1, Table S1: Chemical composition of *Origanum majorana* (marjoram) essential oil [13]; Table S2: Chemical composition of *Rosmarinus officinalis* (rosemary) essential oil [13].
