# Supplementary material for: Target Enzymes of Origanum majorana and Rosmarinus officinalis Essential Oils in Black Cutworm (Agrotis ipsilon): In Vitro and In Silico Studies
Source: Insects. 2024 Jun 28;15(7):483. doi: 10.3390/insects15070483 (PMC11276864; doi:10.3390/insects15070483)
Supplement: Supplementary file 1 [file insects-15-00483-s001.zip › insects-3040820-supplementary.pdf]

Supplementary Data

Table S1. Chemical composition of ***Origanum majorana*** (marjoram) essential oil. **[1]**

RT	Area %	Compound	MF	MS fragmentations
5.13	1.75	Sabinene	876	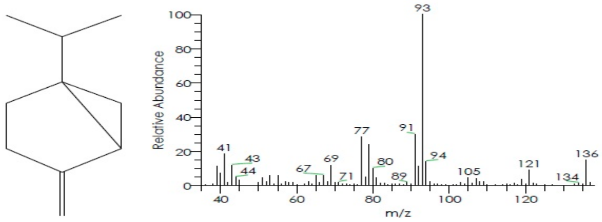
6.25	11.72	o-Cymene	929	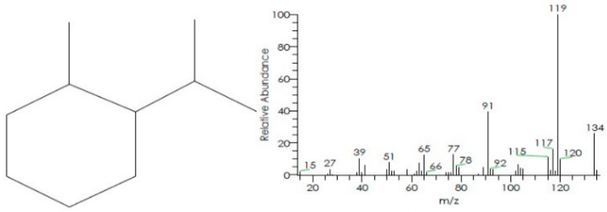
6.86	4.21	γ-Terpinene	909	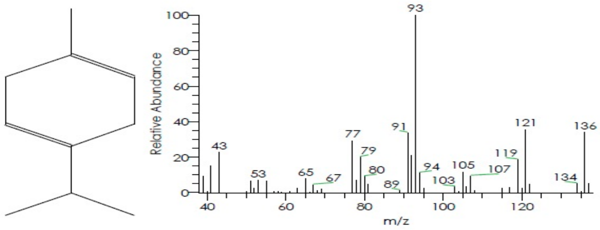
7.21	8.85	cis-β-terpineol	942	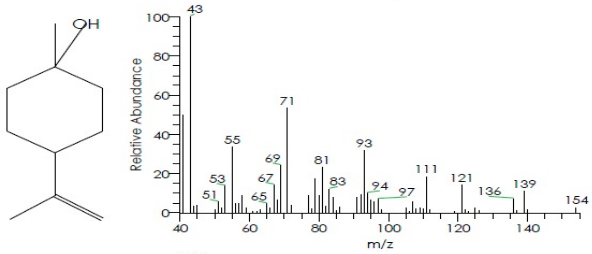
7.90	18.40	(Z)-sabinene hydrate (cis-4-thujanol)	950	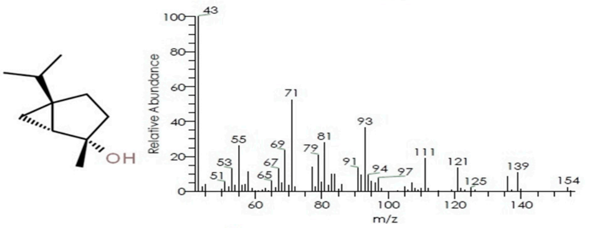
8.42	2.95	trans-para-2-menthen-1-ol	939	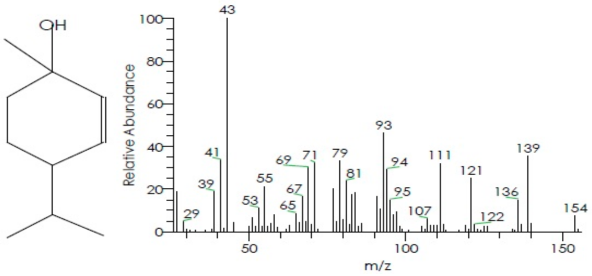

Table 1 continoud

8.86	1.70	cis-para-2-menthen-1-ol	905	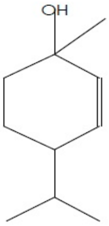	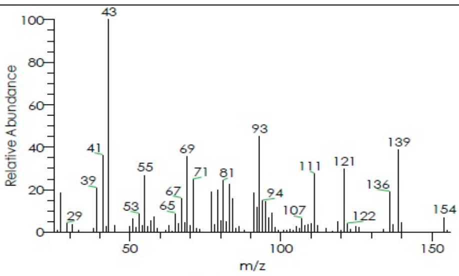
9.62	39.35	Terpinen-4-ol	926	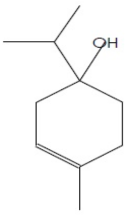	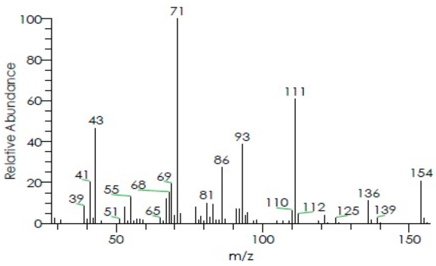
10.05	3.12	α-Terpineol	865	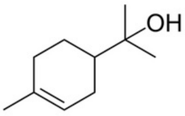	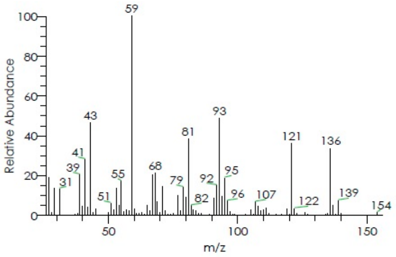
11.04	3.47	Linalyl acetate	860	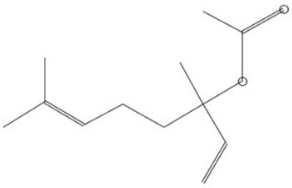	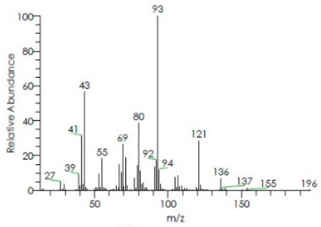
12.08	1.85	iso-3-thujyl acetate	827	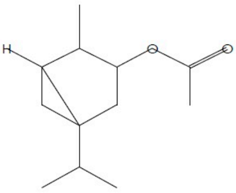	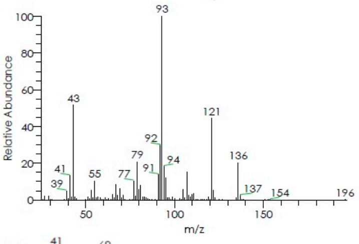
14.66	2.62	Caryophyllene		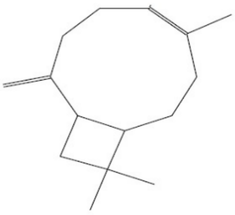	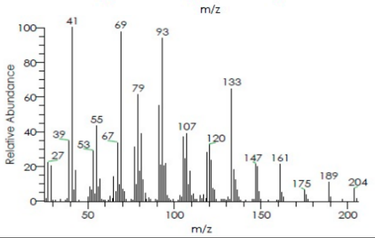

MF: Match factor

Table S2. Chemical composition of ***Rosmarinus officinalis*** (Rosemary) essential oil. **[1]**

RT	Area %	Compound	MF	MS fragmentations
4.36	34.29	α-Pinene	928	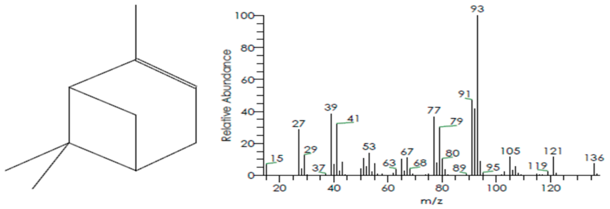
4.70	13.58	Sabinene	823	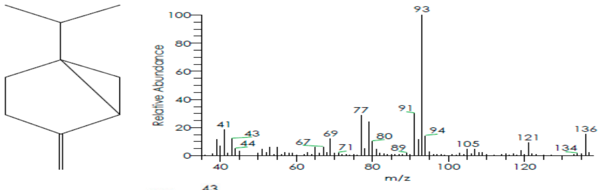
6.21	29.70	Eucalyptol	884	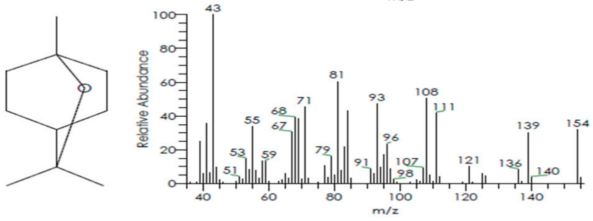
8.72	14.59	Camphor	905	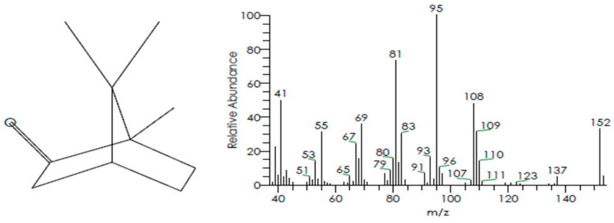
9.43	7.83	Borneol	852	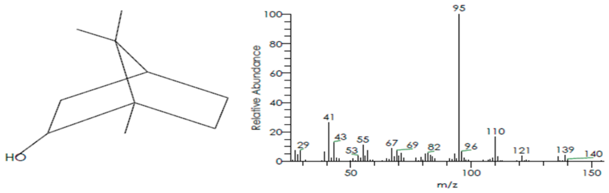

MF: Match factor

References

1. Awad, M.; Hassan, N.; Alfuhaid, N.; Amer, A.; Salem, M.; Fónagy, A.; Moustafa, M. Insecticidal and Biochemical Impacts with Molecular Docking Analysis of Three Essential Oils against Spodoptera Littoralis (Lepidoptera: Noctuidae). *Crop Protection* **2024**, *180*, 106659, doi:10.1016/j.cropro.2024.106659.
